# Predicting Epidemic Risk from Past Temporal Contact Data

**DOI:** 10.1371/journal.pcbi.1004152

**Published:** 2015-03-12

**Authors:** Eugenio Valdano, Chiara Poletto, Armando Giovannini, Diana Palma, Lara Savini, Vittoria Colizza

**Affiliations:** 1 INSERM, UMR-S 1136, Institut Pierre Louis d’Epidémiologie et de Santé Publique, F-75013 56 bd Vincent Auriol—CS 81393-75646 Paris Cedex 13, France; 2 Sorbonne Universités, UPMC Univ Paris 06, UMR-S 1136, Institut Pierre Louis d’Epidémiologie et de Santé Publique, F-75013 56 bd Vincent Auriol—CS 81393 - 75646 Paris Cedex 13, France; 3 Istituto Zooprofilattico Sperimentale Abruzzo-Molise G. Caporale Campo Boario, 64100 Teramo, Italy; 4 ISI Foundation Via Alassio 11/c, 10126 Torino, Italy; CNRS, FRANCE

## Abstract

Understanding how epidemics spread in a system is a crucial step to prevent and control outbreaks, with broad implications on the system’s functioning, health, and associated costs. This can be achieved by identifying the elements at higher risk of infection and implementing targeted surveillance and control measures. One important ingredient to consider is the pattern of disease-transmission contacts among the elements, however lack of data or delays in providing updated records may hinder its use, especially for time-varying patterns. Here we explore to what extent it is possible to use past temporal data of a system’s pattern of contacts to predict the risk of infection of its elements during an emerging outbreak, in absence of updated data. We focus on two real-world temporal systems; a livestock displacements trade network among animal holdings, and a network of sexual encounters in high-end prostitution. We define the node’s loyalty as a local measure of its tendency to maintain contacts with the same elements over time, and uncover important non-trivial correlations with the node’s epidemic risk. We show that a risk assessment analysis incorporating this knowledge and based on past structural and temporal pattern properties provides accurate predictions for both systems. Its generalizability is tested by introducing a theoretical model for generating synthetic temporal networks. High accuracy of our predictions is recovered across different settings, while the amount of possible predictions is system-specific. The proposed method can provide crucial information for the setup of targeted intervention strategies.

## Author Summary

Following the emergence of a transmissible disease epidemic, interventions and resources need to be prioritized to efficiently control its spread. While the knowledge of the pattern of disease-transmission contacts among hosts would be ideal for this task, the continuously changing nature of such pattern makes its use less practical in real public health emergencies (or otherwise highly resource-demanding when possible). We show that in such situations critical knowledge to assess the real-time risk of infection can be extracted from past temporal contact data. An index expressing the conservation of contacts over time is proposed as an effective tool to prioritize interventions, and its efficiency is tested considering real data on livestock movements and on human sexual encounters.

## Introduction

Being able to promptly identify who, in a system, is at risk of infection during an outbreak is key to the efficient control of the epidemic. The explicit pattern of potential disease-transmission contacts has been extensively used to this purpose in the framework of theoretical studies of epidemic processes, uncovering the role of the pattern’s properties in the disease propagation and epidemic outcomes [1, 2, 3, 4, 5, 6, 7, 8]. These studies are generally based on the assumption that the entire pattern of contacts can be mapped out or that its main properties are known. Although such knowledge would be a critical requirement to conduct risk assessment analyses in real-time, which need to be based on the updated and accurate description of the contacts relevant to the outbreak under study [9], it can hardly be obtained in reality. Given the lack of such data, analyses generally refer to the most recent available knowledge of contact data, implicitly assuming a non-evolving pattern.

The recent availability of time-resolved data characterizing connectivity patterns in various contexts [10, 11, 12, 13, 14, 15, 16, 17, 18, 19, 20, 21, 22] has inevitably weakened the non-evolving assumption, bringing new challenges to the assessment of nodes’ epidemic risk. Traditional centrality measures used to identify vulnerable elements or influential spreaders for epidemics circulating on static networks [1, 2, 4, 23, 24, 25, 26, 27, 28, 29, 30] are unable to provide meaningful information for their control, as these quantities strongly fluctuate in time once computed on the evolving networks [19, 31]. An element of the system may thus act as *superspreader* in a past configuration of the contact network, having the ability to potentially infect a disproportionally larger amount of secondary contacts than other elements [32], and then assume a more peripheral role in the current pattern of contact or even become isolated from the rest of the system [19]. If the rules driving the change of these patterns over time are not known, what information can be extracted from past contact data to infer the risk of infection for an epidemic unfolding on the current (unknown) pattern?

Few studies have so far tried to answer this question by exploiting temporal information to control an epidemic through targeted immunization. They are based on the extension to temporal networks [33, 34] of the so-called acquaintance immunization protocol [4] introduced in the framework of static networks that prescribes to vaccinate a random contact of a randomly chosen element of the system. In the case of contacts relevant for the spread of sexually transmitted infections, Lee et al. showed that the most efficient protocol consists in sampling elements at random and vaccinating their latest contacts [33]. The strategy is based on local information gathered from the observation and analysis of past temporal data, and it outperforms static-network protocols. Similar results are obtained for the study of face-to-face contact networks relevant for the transmission of acute respiratory infections in a confined setting, showing in addition that a finite amount of past network data is in fact needed to devise efficient immunization protocols [34].

The aim of these studies is to provide general protocols of immunization over all possible epidemiological conditions of the disease (or class of diseases) under study. For this reason, protocols are tested through numerical simulations and results are averaged over starting seeds and times to compare their performance. Previous work has however shown that epidemic outcomes may strongly depend on the temporal and geographical initial seed of the epidemic [35], under conditions of large dynamical variability of the network and absence of stable structural backbones [19]. Our aim is therefore to focus on a specific epidemiological condition relative to a given emerging outbreak in the population, resembling a realistic situation of public health emergency. We focus on the outbreak initial phase prior to interventions when facing the difficulty that some infected elements in the population are not yet observed. The objective is to assess the risk of infection of nodes to inform targeted surveillance, quarantine and immunization programs, assuming the lack of knowledge of the explicit contact pattern on which the outbreak is unfolding. Knowledge is instead gathered from the analysis of the full topological and temporal pattern of past data (similarly to previous works [33, 34]), coupled, in addition, with epidemic spreading simulations performed on such data under the same epidemiological conditions of the outbreak under study. More specifically, we propose an egocentric view of the system and assess whether and to what extent the node’s tendency of repeating already established contacts is correlated with its probability of being reached by the infection. Findings obtained on past available contact data are then used to predict the infection risk in the current unknown epidemic situation. We apply this risk assessment analysis to two large-scale empirical datasets of temporal contact networks—cattle displacements between premises in Italy [19, 36], and sexual contacts in high-end prostitution [16]—and evaluate its performance through epidemic spreading simulations. We also introduce a model to generate synthetic time-varying networks retaining the basic mechanisms observed in the empirical networks considered, in order to explain the results obtained by the proposed risk assessment strategy within a general theoretical framework.

## Results and Discussion

The cattle trade network is extracted from the complete dataset reporting on time-resolved bovine displacements among animal holdings in Italy [19, 36] for the period 2006–2010, and it represents the time-varying contact pattern among the 215,264 premises composing the system. The sexual contact network represents the connectivity pattern of sexual encounters extracted from a Web-based Brazilian community where sex buyers provide time-stamped rating and comments on their experiences with escorts [16].

**Fig 1:**
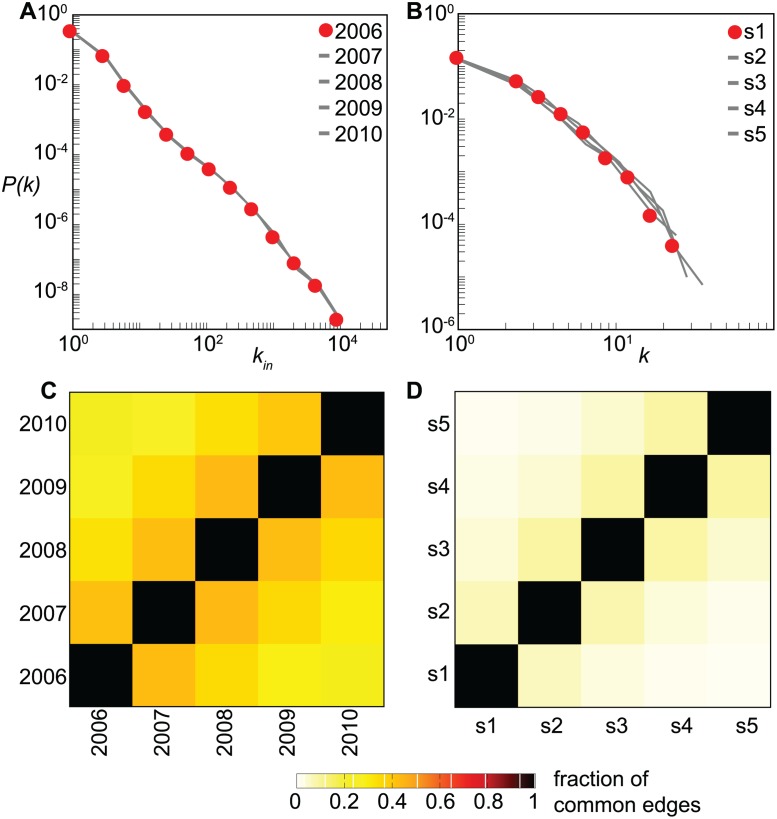
Structural and temporal properties of the cattle trade network and of the sexual contact network. (*A*), (*B*): premises in-degree distributions in the cattle trade network and sex customers degree distribution in the sexual contact network, respectively. Distributions for different configurations of the networks are superimposed in both cases. (*C*), (*D*): fraction of common edges contained in two configurations of the network, for the cattle trade network and the sexual contact network, respectively. In (*B*), (*D*) *s* stands for semester, the aggregation interval of each configuration.

The five-years data of the livestock trade network show that stationary properties at the global level co-exist with an active non-trivial local dynamics. The probability distributions of several quantities measured on the different yearly networks are considerably stable over time, as e.g. shown by the in-degree distribution reported in Fig. 1A, where the in-degree of a farm measures the number of premises selling cattle to that farm. These features, however, result from highly fluctuating underlying patterns of contacts, never preserving more than 50% of the links from one yearly configuration to another (Fig. 1C), notwithstanding the seasonal annual pattern due to repeating cycles of livestock activities [37, 38] (see S1 Text). Similar findings are also obtained for the sexual contact network (Fig. 1B-D), where the lack of an intrinsic cycle of activity characterizing the system leads to smaller values of the overlap between different configurations (< 10%). In this case we consider semi-annual configurations, an arbitrary choice that allows us to extract six network configurations in a timeframe exhibiting an approximately stationary average temporal profile of the system, after discarding an initial transient time period from the data [16]. Different time-aggregating windows are also considered (see the Materials and Methods section and S1 Text for additional details).

### Loyalty

The observed values of the overlap of the time-resolved contact networks in terms of the number of links preserved are a measure of the degree of memory contained in the system. This is the outcome of the temporal activity of the elements of the system that reshape up to 50% or 90% of the contacts of the network (in the cattle trade case and in the sexual contact case, respectively), through nodes’ appearance and disappearance, and neighborhood restructuring. By framing the problem in an egocentric perspective, we can explore the behavior of each single node of the system in terms of its tendency to remain active in the system and re-establish connections with the same partners vs. the possibility to change partners or make no contacts. We quantitatively characterize this tendency by introducing the *loyalty* *θ*, a quantity that measures the fraction of preserved neighbors of a node for a pair of two consecutive network configurations in time, *c*−1 and *c*. If we define ${𝒱}_{i}^{c-1}$ as the set of neighbors of node *i* in configuration *c*−1, then $\theta_{i}^{c-1,c}$ is given by the Jaccard index between ${𝒱}_{i}^{c-1}$ and ${𝒱}_{i}^{c}$:

$$\begin{array}{c} \theta_{i}^{c-1,c}=\frac{\left|{𝒱}_{i}^{c-1}\cap {𝒱}_{i}^{c}\right|}{\left|{𝒱}_{i}^{c-1}\cup {𝒱}_{i}^{c}\right|}\;. \end{array}$$ (1)

Loyalty takes values in the interval [0, 1], with *θ* = 0 indicating that no neighbors are retained, and *θ* = 1 that exactly the same set of neighbors is preserved (${𝒱}_{i}^{c-1}={𝒱}_{i}^{c}$). It is defined for discrete time windows (*c*, *c*+1) and in general it depends on the aggregation interval chosen to build network configurations.

In case the network is directed, as for example the cattle trade network, *θ* can be equivalently computed on the set ${𝒱}_{in,i}^{c}$ of incoming contacts or on the set of neighbors of outgoing connections, ${𝒱}_{out,i}^{c}$, depending on the system-specific interpretation of the direction and on the interest in one phenomenon or the opposite. This measure originally finds its inspiration in the study of livestock trade networks, where a directed connection from holding *A* to holding *B* indicates that *B* purchased a livestock batch from *A*, which was then displaced along the link direction *A* → *B*. If we compute *θ* on the incoming contacts of the cattle trade network, we thus quantify the propensity of each farmer to repeat business deals with the same partners when they purchase their cattle. This concept is at the basis of many loyalty or fidelity programs that propose explicit marketing efforts to incentivize the reinforcement of loyal buying behavior between a purchasing client and a selling company [39], and corresponds to a principle of exclusivity in selecting economic and social exchange partners [40, 41]. Analogously, in the case of the sexual contact network we consider the point of view of sex buyers. Formally, our methodology can be carried out with the opposite point of view, by considering out-degrees with loyalties being computed on out-neighbors. Our choice is arbitrary and inspired by the trade mechanism underlying the network evolution.

Other definitions of similarity to measure the loyal behavior of a node are also possible. In S1 Text we compare and discuss alternative choices. For the sake of clarity all symbols and variables used in the article are reported in Table 1. Finally, other mechanisms different from fidelity strategies may be at play that result in the observed behavior of a given node. In absence of additional knowledge on the behavior underlying the network evolution, we focus on the loyalty *θ* to explore whether it can be used as a possible indicator for infection risk, as illustrated in the following subsection.

**Table 1:** List of variables and their description.

**Notation**	**Description**
*c*	index for network configurations
*θ* or $\theta_{i}^{c-1,c}$	loyalty of node *i* between configurations *c*−1, *c*
${𝒱}_{in,i}^{c},{𝒱}_{out,i}^{c}$	set of in(out)-neighbors of *i* in config *c*
*L*, *D*	loyalty classes (loyal, disloyal)
*ϵ*	loyalty threshold
*s*	epidemic seed
*τ*	duration of the outbreak early stage
${𝓘}_{s}^{c}$	set of infected nodes for outbreak starting from *s* in config *c*
$\pi_{D}^{c-1,c}(s),\pi_{L}^{c-1,c}(s)$	infection potentials for class *D* (*L*) computed for seed *s* between configs *c*−1, *c*
*k*	degree (in-degree for the cattle trade network)
$T_{DD}^{c}(k)$, $T_{DL}^{c}(k)$, $T_{LD}^{c}(k)$, $T_{LL}^{c}(k)$	transition probability from one loyalty class to another
$\rho_{i}^{c}$	epidemic risk for node *i* in config *c*
${𝓘}_{s,h}^{c},{𝓘}_{s,l}^{c}$	set of infected nodes with high(low) epidemic risk
*P*_*h*_, *P*_*l*_	probability of a high(low) risk node to be infected
*ν* = *P*_*h*_/*P*_*l*_	risk ratio between *P*_*h*_, *P*_*l*_, measure of accuracy
$\omega_{s}^{c-1,c}$	predictive power (fraction of infected nodes for which it is possible to compute the epidemic risk)
*b*, *d*	node probability of becoming active or inactive
*p*_*α*_	node probability of keeping an in-neighbor
*α*	number of kept in-neighbors
*β*_*in*_	number of new in-neighbors
*β*_*out*_	number of new out-neighbors
*γ*, *δ*	exponents of the distributions of *β*_*in*_, *β*_*out*_

**Fig 2:**
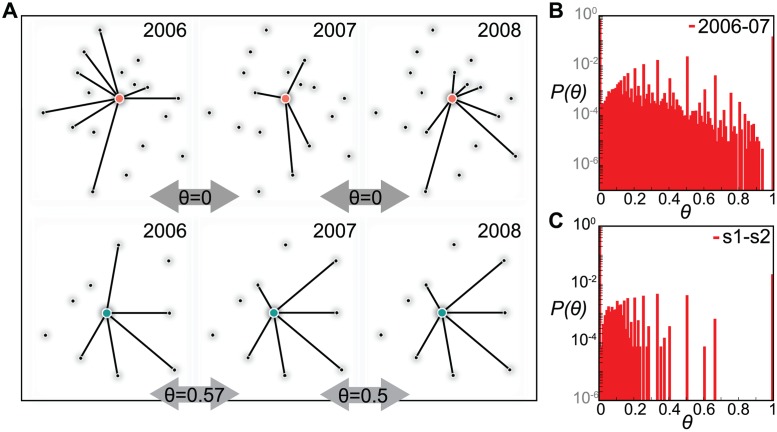
Loyalty. (*A*) Visualization of the neighborhood of two different farms in the cattle trade network (orange node, characterized by low loyalty, and green node, characterized by high loyalty) and corresponding loyalty values computed on three consecutive configurations (2006, 2007, 2008). (*B*), (*C*): Loyalty distributions in the cattle trade network and in the sexual contact network, respectively. Histograms refer to the first pair of consecutive configurations for visualization purposes, all other distributions being reported in S1 Text and showing stability across time.

The distributions of loyalty values, though of different shapes across the two datasets, display no considerable variation moving along consecutive pairs of configurations of each dataset (Fig. 2A-B and S1 Text), once again indicating the overall global stability of system’s properties in time and confirming the results observed for the degree. A diverse range of behaviors in establishing new connections vs. repeating existing ones is observed, similarly to the stable or exploratory strategies found in human communication [42]. Two pronounced peaks are observed for *θ* = 0 and *θ* = 1, both dominated by low degree nodes for which few loyalty values are allowed, given the definition of Eq. (1) (see S1 Text for the dependence of *θ* on nodes’ degree and its analytical understanding). The exact preservation of the neighborhood structure (*θ* = 1) is more probable in the cattle trade network than in the sexual contact network (*P*(*θ* = 1) being one order of magnitude larger), in agreement with the findings of a higher system-wide memory reported in Fig. 1. Moreover, the cattle trade network exhibits the presence of high loyalty values (in the range *θ* ∈ [0.7, 0.9]), differently from the sexual contact network where *P*(*θ*) is always equal to zero in that range except for one pair of consecutive configurations giving a positive probability for *θ* = 0.8. Farmers in the cattle trade network thus display a more loyal behavior in purchasing cattle batches from other farmers with respect to how sex buyers establish their sexual encounters in the analyzed sexual contact dataset.

For the sake of simplification, we divide the set of nodes composing each system into the subset of *loyal nodes* having *θ* greater than a given threshold *ϵ*, and the subset of *disloyal nodes* if instead *θ* < *ϵ*. We call hereafter these classes as *loyalty statuses L* and *D*, respectively, and we will later discuss the role of the chosen value for *ϵ*.

### Epidemic simulations and risk of infection

Both networks under study represent substrates offering potential opportunities for a pathogen to diffuse in the corresponding populations. Sexually transmitted infections spread among the population of individuals through sexual contacts [43, 44], whereas livestock infectious diseases (e.g. Foot-and-mouth disease [45], Bluetongue virus [46], or BVD [47]) can be transmitted from farm to farm mediated by the movements of infected animals (and vectors, where relevant), potentially leading to a rapid propagation of the disease on large geographical scales.

As a model for disease-transmission on the network of contacts we consider a discrete-time Susceptible-Infectious compartmental approach [48]. No additional details characterizing the course of infection are considered here (e.g. recovery dynamics), as we focus on a simplified theoretical picture of the main mechanisms of pathogen diffusion and their interplay with the network topology and time-variation, for the prediction of the risk of infection. The aim is to provide a general and conceptually simple framework, leaving to future studies the investigation of more detailed and realistic disease natural histories.

At each time step, an infectious node can transmit the disease along its outgoing links to its neighboring susceptible nodes that become infected and can then propagate the disease further in the network. Here, we consider a deterministic process for which the contagion occurs with probability equal to 1, as long as there exist a link connecting the infectious node to a susceptible one. Although a crude assumption, this allows us to simplify the computational aspects while focusing on the risk prediction. The corresponding stochastic cases exploring lower probabilities of transmission per link are reported in S1 Text.

We focus on the early phase of the spreading simulations, defined as the set of nodes infected up to simulation time step *τ* = 6. This choice allows us to study invasion stage only, while the epidemic is no more trivially confined to the microscopic level. Additional choices for *τ* have been investigated showing that they do not alter our findings (see S1 Text). Network configurations are kept constant during outbreaks, assuming diseases spread faster than network evolution, at least during their invasion stage. Examples of incidence curves obtained by the simulations are reported in S1 Text.

Livestock disease spread is often modeled by assuming that premises are the single discrete units of the spreading processes and neglecting the possible impact of within-farm dynamics [49]. This is generally considered in the study of highly contagious and rapid infections, and corresponds to regarding a farm as being infected as soon as it receives the infection from neighboring farms following the transport of contagious animals. Under this assumption, both case studies can be analyzed in terms of networks of contacts for disease transmission. In addition, for sake of simplicity, we do not take into account the natural definition of link weights on cattle network, representing the size of the moved batches. In S1 Text we generalize our methodology to the weighted case, including a weighted definition of loyalty, reaching results similar to the unweighted case.

We consider an emerging epidemic unfolding on a network configuration *c* and starting from a single node (seed *s*), where the rest of the population of nodes is assumed to be initially susceptible. The details on the simulations are reported in the Material and Methods section. We define ${𝓘}_{s}^{c}$ the set of nodes infected during the early stage invasion. In order to explore how the network topology evolution alters the spread of the disease, we consider an outbreak unfolding on the previous configuration of the system, *c*−1, and characterized by the same epidemiological conditions (same epidemic parameters and same initial seed *s*). By comparing the set of infected nodes ${𝓘}_{s}^{c-1}$ obtained in configuration *c*−1 to ${𝓘}_{s}^{c}$, we can assess changes in the two sets and how these depend on the nodes’ loyalty. We define a node’s infection potential $\pi_{L}^{c-1,c}(s)$ ($\pi_{D}^{c-1,c}(s)$) measuring the probability that a node will be infected in configuration *c* by an epidemic starting from seed *s*, given that it was infected in configuration *c*−1 under the same epidemiological conditions and provided that its loyalty status is *L* (*D*):

$$\begin{array}{cc} & \pi_{L}^{c-1,c}\left(s\right)\overset{def}{=}\text{Prob}\left[i\in {ℐ}_{s}^{c}\;|\;i\in {ℐ}_{s}^{c-1}\;\text{and}\;i\in \left\{L\right\}\right],\\ & \pi_{D}^{c-1,c}\left(s\right)\overset{def}{=}\text{Prob}\left[i\in {ℐ}_{s}^{c}\;|\;i\in {ℐ}_{s}^{c-1}\;\text{and}\;i\in \left\{D\right\}\right], \end{array}$$

where *i* is a node of the system. *π*_*L*_ and *π*_*D*_ thus quantify the effect of the temporal stability of the network at the local level (loyalty of a node) on the stability of a macroscopic process unfolding on the network (infection). They depend on the seed chosen for the start of the epidemic, on the pair (*c*−1, *c*) of network configurations considered along its evolution, and also on the threshold value *ϵ* assumed for the definition of the loyalty status of the nodes.

**Fig 3:**
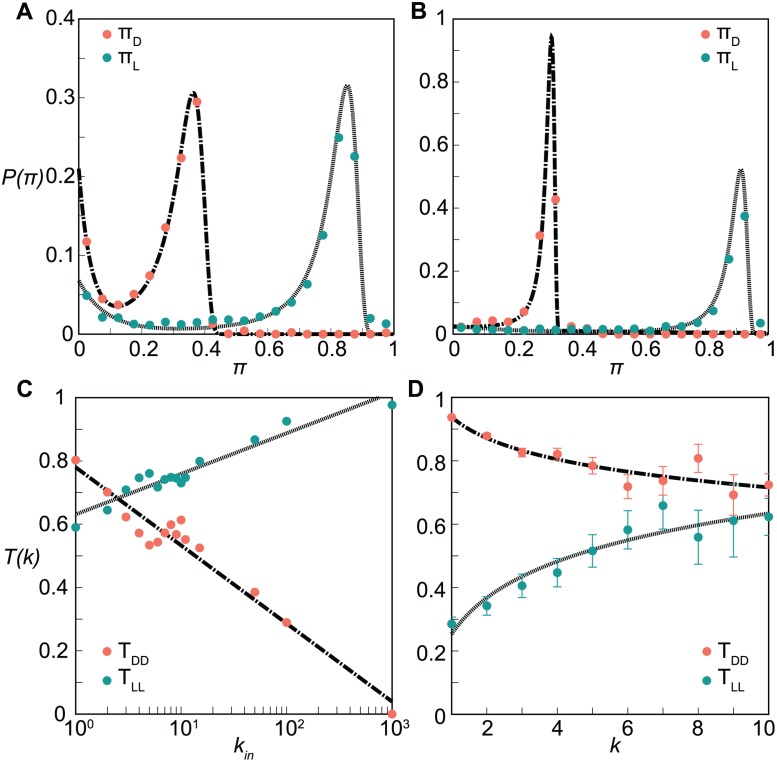
Infection potentials and loyalty transitions. (*A*), (*B*): Probability distributions of the infections potentials for loyal (*π*_*L*_, green) and disloyal nodes (*π*_*D*_, orange), for the cattle trade network and the sexual contact network, respectively. Loyalty is set with a threshold *ϵ* = 0.1. Dashed lines show the fit with a Landau+exponential model (see Material and Methods). (*C*), (*D*): Loyalty transition probabilities between loyal statuses (*T*_*LL*_(*k*), green) and disloyal statuses (*T*_*DD*_(*k*), orange) as functions of the degree *k* of the node, for the cattle trade network and the sexual contact network, respectively. Dashed lines represent the logarithmic models: *T*_*DD*_(*k*) = 0.78−0.11log *k*, and *T*_*LL*_(*k*) = 0.63+0.06log *k* for the cattle trade network; *T*_*DD*_(*k*) = 0.94−0.10log *k*, and *T*_*LL*_(*k*) = 0.25+0.17log *k* for the sexual contact network. Transition probabilities are computed as frequencies in the datasets under study. The error bars here represent one binomial standard deviation from these frequencies. In (*C*) the error bars are smaller than the size of the points. A single pair of configurations is considered here as example; the behavior observed is the same for all the pair of configurations.

By exploring all seeds and computing the infection potentials for different couples of years, we obtain sharply peaked probability distributions of *π*_*L*_ and *π*_*D*_ around values that are well separated along the *π* axis. Results are qualitatively similar in both cases under study, with peaks reached for *π*_*L*_/*π*_*D*_ ≃ 2.5 in the cattle trade network and *π*_*L*_/*π*_*D*_ ≃ 3 in the sexual trade network (Fig. 3A-B). An observed infection in *c*−1, based on the knowledge of the epidemiological conditions and no information on the network evolution, is an indicator of an infection risk for the same epidemic in *c* more than twice larger for loyal farms with respect to disloyal farms. Analogously, loyal sex buyers have a threefold increase in their infection potential with respect to individuals having a larger turnover of partners. Remarkably, small values of loyalty threshold *ϵ* are able to correctly characterize the loyal behavior of nodes with status *L*. Results shown in Fig. 3A-B are obtained for *ϵ* = 0.1. Findings are however robust against changes in the choice of the threshold value, as this is induced by the peculiar bimodal shape of the probability distribution curves for the loyalty (see S1 Text). This means that intermediate values of the local stability of the nodes (i.e. *θ* > *ϵ*) imply that a possible risk of being infected is strongly stable, regardless of the dynamics of the network evolution. Valid for all possible seeds and epidemiological conditions, this result indicates that the loyalty of a node can be used as an indicator for the node’s risk of infection, which has important implication for the spreading predictability in case an outbreak emerges.

These results are obtained for temporally evolving networks where no further change induced by the epidemic is assumed to occur. Focusing on the initial stage of the outbreak, we disregard the effect of interventions (e.g. social distancing, quarantine of infectious nodes, movements bans) or of adaptive behavior following awareness [37, 50, 51, 52, 53, 54]. Such assumption relies on the study’s focus on the initial stage of the epidemic that may be characterized by a silent spreading phase with propagation occurring before the alert or outbreak detection takes place; or, following an alert, by a contingent delay in the implementation of intervention measures.

### Risk assessment analysis

The observed relationship between loyalty and infection potential can be used to define a strategy for the risk assessment analysis of an epidemic unfolding on an unknown networked system at present time, for which we have however information on its past configurations. This may become very useful in practice even in the case of complete datasets, as for example with emerging outbreaks of livestock infectious diseases. Data on livestock movements are routinely collected following European regulations [55], however they may not be readily available in a real-time fashion upon an emergency, and a certain delay may thus be expected. Following an alert for an emerging livestock disease epidemic, knowledge of past network configurations may instead be promptly used in order to characterize the loyalty of farmers, simulate the spread of the disease on past configurations and thus provide the expected risk of infection for the farms under the ongoing outbreak. The general scheme of the strategy for the risk assessment analysis is composed of the following steps, assuming that the past network configurations {*c*−*n*, …, *c*−1, *c*} are known and that the epidemic unfolds on the unknown configuration *c*+1:

1. identify the seed *s* of the ongoing epidemic;
2. characterize the loyalty of the nodes from past configurations by computing $\theta_{i}^{c-1,c}$ from Eq. (1);
3. predict the loyalty of the nodes for the following unknown configuration *c*+1: $\theta_{i}^{c,c+1}$;
4. simulate the spread of the epidemic on the past configuration *c* under the same epidemiological conditions of the ongoing outbreak and identify the infected nodes ${𝓘}_{s}^{c}$;
5. compute the node epidemic risk for nodes in statuses *L* and *D*.

This strategy enables the assessment of the present infection risk (i.e. on configuration *c*+1) for all nodes hit by the simulated epidemic spreading on past configuration *c* (${𝓘}_{s}^{c}$), not knowing their present pattern of contacts. It is based on configurations from *c*−*n* to *c* as they are all used to build the probability distributions needed to train our approach. In the cases under study such distributions are quite stable over time so that a small set of configurations ({*c*−2, *c*−1, *c*}) was shown to be enough.

To make the above strategy operational, we still need to determine how we can exploit past data to predict the evolution of the loyalty of a node in future configurations (step 3) and use this information to compute nodes epidemic risk (point 5). As with all other variables characterizing the system, indeed, also *θ* may fluctuate from a pair of configurations (*c*−1, *c*) to another, as nodes may alter their loyal behavior over time, increasing or decreasing the memory of the system across time. Without any additional knowledge or prior assumption on the dynamics driving the system, we measure from available past data the probabilities of (dis)loyal nodes staying (dis)loyal across consecutive configurations, or conversely, of changing their loyalty status. This property can be quantified in terms of probabilities of transition across loyalty statuses. We thus define $T_{LL}^{c}(k)$ as the probability that a node with degree *k* being loyal between configurations *c*−1 and *c* will stay loyal one step after (*c*, *c*+1). It is important to note the explicit dependence on the degree *k* of the node (here defined at time *c*), which may increase or decrease following neighborhood reshaping (it may also assume the value *k* = 0 if the node becomes inactive in configuration *c*). Analogously, $T_{DD}^{c}(k)$ is the probability of remaining disloyal. The other two possible transition probabilities are easily obtained as *T*_*LD*_ = 1−*T*_*LL*_ and *T*_*DL*_ = 1−*T*_*DD*_.

Fig. 3C-D show the transition probabilities of maintaining the same loyalty status calculated on the two empirical networks for *ϵ* = 0.1. Stability in time and non-trivial dependences on the degree of the node are found for both networks. In the cattle trade network, loyal farmers tend to remain loyal with a rather high probability (*T*_*LL*_ > 0.6 for all *k*_*in*_ values). In addition, this probability markedly increases with the degree, reaching *T*_*LL*_ ≃ 1 for the largest values of *k*_*in*_. Interestingly, the probability that a disloyal farmer stays disloyal the following year dramatically decreases with the degree, reaching 0 in the limit of large degree. Among the farmers who purchase cattle batches from a large number of different premises, loyal ones have an increased chance to establish business deals with the same partners the following year, whereas previously disloyal ones will more likely turn to being loyal.

A similar qualitative dependence on the degree is also found in the sexual contact network, however in this case the probability of remaining disloyal is always very high (*T*_*DD*_ > 0.7) even for high degrees. *T*_*LL*_ shows a relatively more pronounced dependence on *k*, ranging from 0.3 (low degree nodes) to 0.6 (high degree nodes). Differently from the farmers behavior, sex buyers display a large tendency to keep a high rate of partners turnover across time. Moreover, the largest probability of preserving sexual partners is obtained when the number of partners is rather large.

Remarkably, in both networks, transition probabilities are found to be stable across time and are well described by logarithmic functions (with parameters depending on the system and on *ϵ*) that can be used to predict the loyalty of nodes in configuration *c*+1 from past data (Fig. 3C-D). With this information, it is then possible to compute the epidemic risk of a node *i* in configuration *c*+1, having degree $k=k_{i}^{c}$ in configuration *c* and known loyalty status {*L*, *D*} between configurations *c*−1 and *c* as follows:

$$\begin{array}{c} \left\{\begin{array}{c} \text{if}\;\text{loyalty}\;\text{class}=D:\;\;\;\;\rho_{i}^{c+1}=\pi_{D}^{c,c+1}\left(s\right)T_{DD}\left(k\right)+\pi_{L}^{c,c+1}\left(s\right)T_{DL}\left(k\right)\;;\\ \text{if}\;\text{loyalty}\;\text{class}=L:\;\;\;\;\rho_{i}^{c+1}=\pi_{D}^{c,c+1}\left(s\right)T_{LD}\left(k\right)+\pi_{L}^{c,c+1}\left(s\right)T_{LL}\left(k\right)\;. \end{array}\right. \end{array}$$ (2)

It is important to note that in our framework the epidemic risk is a node property, and not a global characteristic of a specific disease.

### Validation

**Fig 4:**
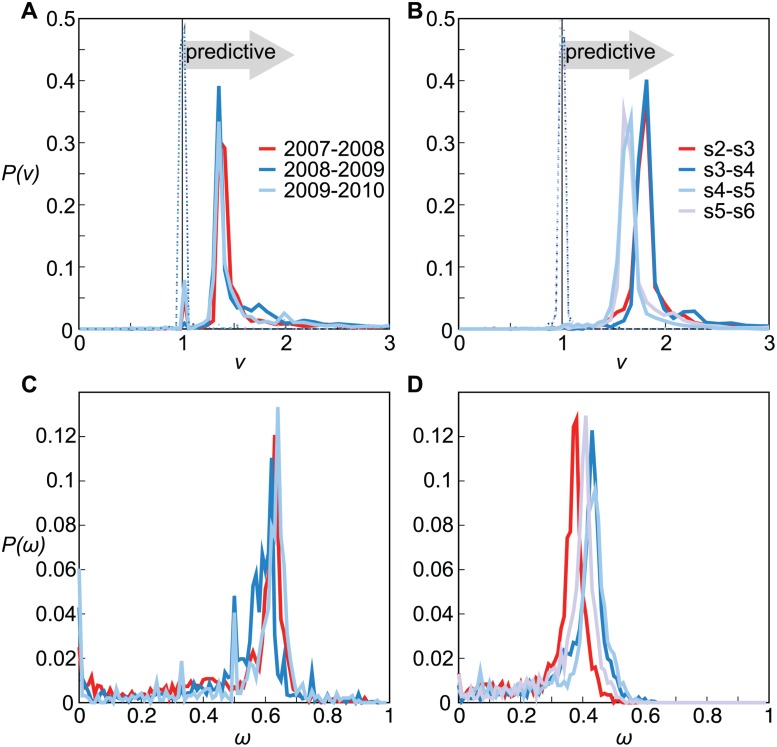
Validation of the risk assessment analysis. (*A*), (*B*): Probability distributions of the risk ratio *ν* for the cattle trade network and the sexual contact network, respectively. Red lines are computed on training sets (2007–08 for cattle and s2-s3 for sexual contacts). The dashed lines peaking around 1 represent a null model based on reshuffling the infection statuses, i.e. randomly permuting the attribute *“actually being infected”* among the nodes for which risk assessment is performed. (*C*), (*D*): Probability distributions of the predictive power *ω* for the cattle trade network and the sexual contact network, respectively.

To validate our strategy of risk assessment, we test our predictions based on past data for the risk of being infected in configuration *c*+1 on the results of an epidemic simulation explicitly performed on the supposedly unknown configuration *c*+1. We consider the set of nodes ${𝓘}_{s}^{c}$ for which we are able to provide risk predictions and divide it into two subsets, according to their predicted risk of infection $\rho_{i}^{c+1}$. We indicate with ${𝓘}_{s,h}^{c}$ the top 25% highest ranking nodes, and with ${𝓘}_{s,l}^{c}$ all the remaining others. We then compute the fraction *P*_*h*_ of nodes in the subset ${𝓘}_{s,h}^{c}$, i.e. predicted at high risk, that belong to the set of infected nodes ${𝓘}_{s}^{c+1}$ in the simulated epidemic aimed at validation. Analogously, *P*_*l*_ measures the fraction of nodes in ${𝓘}_{s,l}^{c}$ that are reached by the infection in the simulation on *c*+1. In other words, *P*_*h*_ (*P*_*l*_) represents the probability for a node having a high (low) risk of infection to indeed get infected. The accuracy of the risk assessment analysis can thus be measured in terms of the relative risk ratio *ν* = *P*_*h*_/*P*_*l*_, where values *ν* ≤ 1 indicate negative or no correlation between our risk predictions and the observed infections, whereas values *ν* > 1 indicate that the prediction is informative. For both networks we find a significant correlation, signaled by the distributions of the relative risk ratio *ν* peaking around values *ν* > 1 (Fig. 4A-B). The peak positions (*ν* ≃ 1.4 and *ν* ≃ 1.7 for cattle and sex, respectively) are remarkably close to the benchmark values represented by the distributions computed on the training sets (red lines in Fig. 4A-B). In addition, the comparison with the distributions from a null model obtained by reshuffling the infection statuses of nodes (dotted curves peaking around *ν* = 1 in Fig. 4A-B) further confirms the accuracy of the approach. Findings are robust against changes of the value used to define ${𝓘}_{s,h}^{c}$ or against alternative definitions of this quantity (see S1 Text).

One other important aspect to characterize is the predictive power of our risk assessment analysis. Our predictions indeed are limited to the set ${𝓘}_{s}^{c}$ of nodes that are reached in the simulation performed on past data, proxy for the future outbreak. If a node is not infected by the simulation unfolding on configuration *c* or it is not active at that given time, our strategy is unable to provide a risk assessment for that node in the future. We can then quantify the predictive power *ω* as the fraction of infected nodes for which we could provide the epidemic risk, i.e. $\omega_{s}^{c,c+1}=|{𝓘}_{s}^{c+1}\cap {𝓘}_{s}^{c}|/|{𝓘}_{s}^{c+1}|$. High values of *ω* indicate that few infections are missed by the risk assessment analysis. Fig. 4C-D display the distributions *P*(*ω*) obtained for the two case studies, showing that a higher predictive power is obtained in the cattle trade network (peak at *ω* ≃ 60%) with respect to the sexual contact network (peak at *ω* ≃ 40%). Our methodology can potentially be applied to a wide range of networks, other than the ones presented here, as shown with the example of human face-to-face proximity networks relevant for the spread of respiratory diseases reported in S1 Text.

We also tested whether our risk measure represents a significant improvement in prediction accuracy with respect to simpler and more immediate centrality measures (namely, the degree). Through a multivariate logistic regression, in S1 Text we show that our definition of node risk is predictor of infection even after adjusting for node degree.

### Memory driven dynamical model

The results of the risk assessment analysis obtained from the application of our strategy to the two empirical networks show qualitatively similar results, indicating that the approach is general enough to provide valuable information on the risk of infection in different settings. The observed differences in the predictive power of the approach are expected to be induced by the different temporal behavior of the two systems, resulting in a different amount of memory in preserving links (Fig. 1) and different loyalty of nodes and their time-variations (Fig. 2 and 3C-D).

In order to systematically explore the role of these temporal features on the accuracy and predictive power of our approach, we introduce a generic model for the generation of synthetic temporal networks. The model is based on a set of parameters that can be tuned to reproduce the empirically observed features of the two networks, i.e.: *(i)* the topological heterogeneity of each configuration of the network described by a stable probability distribution (Fig. 1A-B); *(ii)* a vital dynamics to allow for the appearance and disappearance of nodes; *(iii)* a tunable amount of memory characterizing the time evolution of the network contacts (Fig. 1C-D). These specific properties differentiate our approach from the previously introduced models that display instantaneous homogeneous properties for network configurations [56, 57, 58, 59], reproduce bursty inter-event time distributions but without the explicit introduction of memory [33, 60, 61] or of its control [58].

**Fig 5:**
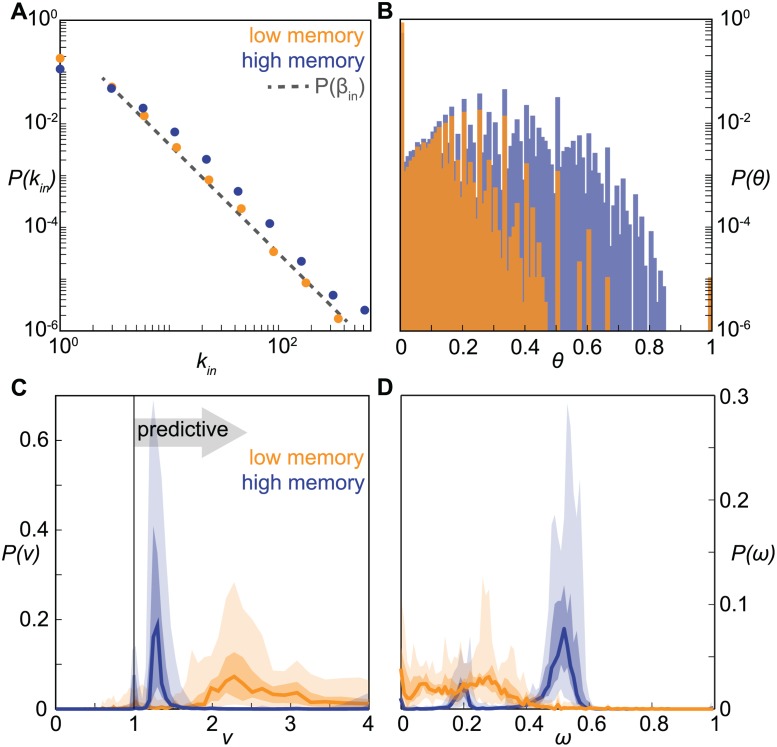
Memory driven dynamical model: model properties and validation of the risk assessment analysis. (*A*): Probability distributions of the node in-degree, in the low (*p*_*α*_ = 0.3) and high memory (*p*_*α*_ = 0.7) regimes. The slope of the distributions does not depend on *p*_*α*_, and it is forced by the exponent *γ* of the *β*_*in*_ distribution (dashed line). (*B*): Probability distributions of the loyalty, in the low and high memory regimes. Distributions are color-coded as in panel (*a*). (*C*): Probability distributions of the risk ratio *ν*, in the low and high memory regimes. Lines represent the median values obtained from 50 realizations of the model; darker and lighter shaded areas represent the 50% and 95% confidence intervals. (*D*): Probability distributions of the predictive power *ω*, in the low and high memory regimes. Medians and confidence intervals are presented as in panel (*C*). Distributions are color-coded as in panel (*A*).

Based on an iterative network generation approach (see Materials and Methods), we can build an arbitrarily large number of configurations of networks with 10^4^ nodes. They are characterized by stable in-degree and out-degree heterogeneous distribution across time (Fig. 5A where high memory and low memory regimes are displayed) and by profiles for the probability distribution of the loyalty as in the empirical networks (Fig. 5B). The number of nodes with zero loyalty can be computed analytically (see Materials and Methods) and it is confirmed by numerical findings (see S1 Text). A high memory regime corresponds to having nodes in the system that display a highly loyal behavior (e.g., *θ* > 0.7), whereas values in the range *θ* ∈ [0.7, 1) are almost absent in a low memory regime, in agreement with the findings of Fig. 2.

Applying the introduced risk assessment analysis to the synthetically generated temporal network, we recover a significant accuracy for both memory regimes (Fig. 5C). Different degrees of memory are however responsible for the fraction of the system for which a risk assessment can be made. In networks characterized by higher memory, the distribution of the predictive power *ω* has a well defined peak, whereas for lower memory it is roughly uniform in the range *ω* ∈ [0, 0.4] (Fig. 5D). Such a regime implies that not enough structure is maintained in the system to control more than 40% of the future infections. Our risk assessment analysis allows therefore accurate predictions across varying memory regimes characterizing the temporal networks, but the degree of memory impacts the amount of predictions that can be made. The model also shows that the analysis is not affected by the choice of the aggregating time window used to define the network configurations [61, 62, 63], as long as the heterogeneous topological features at the system level and the heterogeneous memory at the node level are kept across aggregation, as observed for the empirical networks under study (see [19] and S1 Text).

## Conclusions

We introduce a simple measure to characterize the amount of memory in the time evolution of a networked system. The measure is local and it is empirically motivated from two case studies relevant for disease transmission. By focusing on the degree of loyalty that each node has in establishing connections with the same partners as time evolves, we are able to connect an egocentric view of the system (the node’s strategy in establishing its neighborhood over time) to the system’s larger-scale properties characterizing the early propagation of an emerging epidemic.

We uncover a non-trivial correlation between the loyalty of a node and its risk of being infected if an epidemic occurs, given fixed epidemiological conditions, and use this to inform a risk assessment analysis applicable to different settings with no information on the network evolution dynamics. A theoretical model generating synthetic time-varying networks allows us to frame the analysis in a more general perspective and disentangle the role of different features. The accuracy of the proposed risk assessment analysis is stable across variations of the temporal correlations of the system, whereas its predictive power depends on the degree of memory kept in the time evolution. The introduced strategy can be used to inform preventive actions in preparation to an epidemic and for targeted control responses during an outbreak emergency, only relying on past network data.

## Methods

### Datasets

The cattle trade network is obtained from the database of the Italian national bovine registry recording all cattle displacements due to trade transactions. We consider animal movements during a 5 years time period, from 2006 to 2010, involving 215,264 premises and 2,973,710 directed links. Nodes may be active or inactive depending whether farms sell/buy cattle in a given timeframe. The cattle network is available as S1 Dataset. From the dataset we have removed slaughterhouses (∼ 1% of the nodes) as they are not relevant for transmission.

The sexual contact network is extracted from an online Brazilian forum where male sex buyers rate and comment on their sexual encounters with female sex sellers [16]. Time-stamped posts are used as proxies for sexual intercourse and multiple entries are considered separately, following previous works [16, 31]. A total of 13,855 individuals establishing 34,509 distinct sexual contacts are considered in the study, after discarding the initial transient of the community growth [16]. Nodes may be active or inactive depending whether individuals use or not the service, and join or quit the community. Six-months aggregating snapshots are chosen. A different aggregating time window of three months has been tested, obtaining similar results (see S1 Text).

### Risk of infection

The distributions of the risk potentials *π*_*L*_ and *π*_*D*_ reported in Fig. 3 are modeled with a sum of Landau distribution and an exponential suppression. This family of functions depending on four parameters (see S1 Text for the specific functional form) was chosen as it well reproduces the distribution profiles of the risk potentials, and it was used to compute the nodes’ epidemic risk. A goodness of fit was not performed, as this choice was automatically validated in the validation analysis performed on the whole prediction approach.

### Memory driven model

The basic iterative network generation approach allows to build configuration *c*+1 from configuration *c* through the following steps:

- vital dynamics: nodes that are inactive in configuration *c* become active in *c*+1 with probability *b*, while active nodes become inactive with probability *d*;
- memory: active nodes maintain same in-neighbors each with probability *p*_*α*_; then they form *β*_*in*_ new in-stubs, where *β*_*in*_ is extracted from a power-law distribution: $P(\beta_{in})∼\beta_{in}^{-\gamma }$;
- out-degree heterogeneity: each node is assigned *β*_*out*_ out-stubs, where *β*_*out*_ is drawn from another power-law distribution: $P(\beta_{out})∼\beta_{out}^{-\delta }$. Then each of the in-stubs is randomly matched to an out-stub.

The total set of nodes is considered to be fixed in time, and nodes may be active (i.e. establishing connections) or inactive (i.e. isolated) in a given configuration. All five parameters *b*, *d*, *γ*, *p*_*α*_, *δ* are assumed constant in time and throughout the network. The amount of memory in the system is tuned by the interplay of the two parameters *p*_*α*_ and *d*. Starting from an arbitrarily chosen initial configuration *c* = 0, simulations show that the system rapidly evolves towards a dynamical equilibrium, and successive configurations can be obtained after discarding an initial transient of time. The parameters values used in the paper are: *N* = 10^4^; *b* = 0.7; *d* = 0.2; *γ* = 2.25; *δ* = 2.75; *p*_*α*_ = 0.3, 0.7. The influence of such parameters on the network properties is examined in S1 Text.

If we denote with *α* the number of neighbors that a given node keeps across two consecutive configurations (*c*−1, *c*), we can express the loyalty simply as:

$$\begin{array}{c} \theta_{i}^{c-1,c}=\frac{\alpha^{c-1,c}}{\left(k_{i}^{c}+\beta_{in}^{c}\right)} \end{array}$$ (3)

where the superscript *c* for *α*, *β*_*in*_ indicate the values used to build configuration *c*. The number of nodes with *θ* = 0 as a function of the degree can be computed analytically: $P(\theta_{c,c+1}=0)=d+(1-d){\left(1-p_{\alpha }\right)}^{k_{c}}$. Similarly, it is possible to compute the probability *f*_*c*, *c*+1_ that a link present in configuration *c* is also present in configuration *c*+1. In the S1 Text we show that *f*_*c*, *c*+1_ ≃ (1−*d*)*p*_*α*_ and confirm this result by numerical simulations.

## Supporting Information

S1 Dataset Cattle trade network dataset. We provide the cattle trade network as yearly edge lists, from 2006 to 2010. The dataset consists in five CSV files (one for each year) compressed in a ZIP archive.
(ZIP)

S1 Text Additional analyses. We provide a description of the seasonal pattern of cattle network (Section 1), a more in-depth characterization of loyalty (Section 2), a comparison between loyalty and other similarity measures (Section 3), the specific modeling function for the infection potential (Section 4), the robustness of the risk assessment procedure to variations in parameters and assumptions (Section 5), further analyses of the memory driven model in terms of analytical results (Section 6) and additional properties (Section 7), an extension of our methodology to take into account transmissibility lower than 1 (Section 8), and links weights (Section 9).
(PDF)
