# Supplementary material for: Predicting Epidemic Risk from Past Temporal Contact Data
Source: PLoS Comput Biol. 2015 Mar 12;11(3):e1004152. doi: 10.1371/journal.pcbi.1004152 (PMC4357450; doi:10.1371/journal.pcbi.1004152)
Supplement: S1 Text — We provide a description of the seasonal pattern of cattle network (Section 1), a more in-depth characterization of loyalty (Section 2), a comparison between loyalty and other similarity measures (Section 3), the specific modeling function for the infection potential (Section 4), the robustness of the risk assessment procedure to variations in parameters and assumptions (Section 5), further analyses of the memory driven model in terms of analytical results (Section 6) and additional properties (Section 7), an extension of our methodology to take into account transmissibility lower than 1 (Section 8), and links weights (Section 9). (PDF) [file pcbi.1004152.s002.pdf]

Supporting Information Text S1

Predicting epidemic risk from past temporal contact data

Eugenio Valdano[a,b], Chiara Poletto[a,b], Armando Giovannini[c], Diana Palma[c], Lara Savini[c], and Vittoria Colizza[a,b,d]

[a]INSERM, UMR-S 1136, Institut Pierre Louis d'Epidémiologie et de Santé Publique, F-75013, Paris, France. [b]Sorbonne Universités, UPMC Univ Paris 06, UMR-S 1136, Institut Pierre Louis d'Epidémiologie et de Santé Publique, F-75013, Paris, France. [c]Istituto Zooprofilattico Sperimentale Abruzzo-Molise G. Caporale, Teramo, Italy. [d]ISI Foundation, Torino, Italy.

1 Seasonal pattern in cattle trade network

Fig. S1 shows the number of active links per month in the cattle trade network. A seasonal pattern is clearly visible: the activity drops during summer months, and peaks during fall. The activity pattern is quite similar from one year to the other.

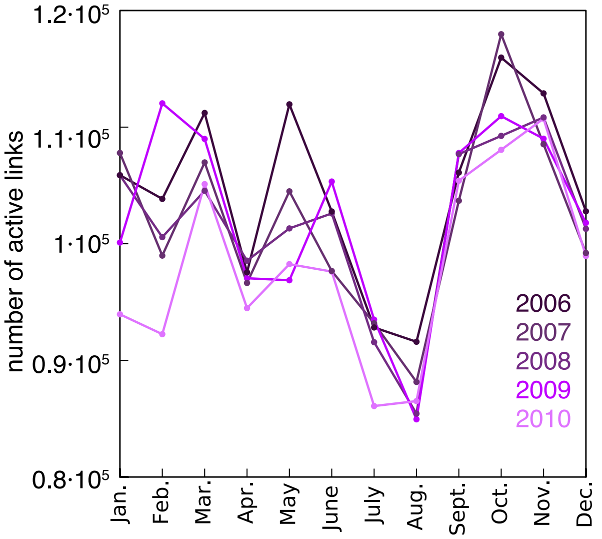

Figure S1: Number of active links per month in cattle trade network. Different colors pertain to different years, in range $(2006 - 2010)$.

2 Loyalty's properties

2.1 Allowed values

In the following we provide an analytical reasoning on the allowed values for the loyalty. θ between configurations c and $c+1$ can be rewritten as

$$\theta = \frac{\alpha}{A-\alpha}, \tag{1}$$

where $\alpha \in \mathbb{N}$ is the number of neighbors retained from c to $c+1$, and $A = k_c + k_{c+1} \in \mathbb{N}$ is the sum of the node's degrees. Clearly, every pair of α', A' for which $\exists q \in \mathbb{N}$ such that $\alpha' = q\alpha$ and $A' = qA$, will give the same θ. Therefore, in order to compute all the possible values of θ, we must restrict ourselves to α, A coprimes: $(\alpha, A) = 1$. Moreover, since θ cannot be higher than 1, we have to impose one further constraint: $\alpha < A/2$. All divisions are to be intended as integer divisions.

For zero loyalty, we have $\theta = 0 \Leftrightarrow \alpha = 0$, for every positive A. If $\theta > 0$, we need to count the number of possible values α, given the constraints discussed above, and given a value for A which is fixed by the node's degrees. For $A \geq 3$, there are $\varphi(A)/2$ coprimes of A and smaller than or equal to $A/2$, as it can be inferred by basic properties of the Euler's totient function φ.

$$n(A) = \begin{cases} 0 & \text{if } A = 1 \\ 1 & \text{if } A = 2 \\ \varphi(A)/2 & \text{if } A \geq 3 \end{cases}, \tag{2}$$

where $n(A)$ counts the number of nonzero allowed values for θ, given a fixed A. In order to compute the total number of allowed θ values in an entire network, we now let A run from 1 to a certain A_{max}, which is of the order of twice the highest degree:

$$\mathcal{N}(A_{max}) = 1 + \sum_{A=1}^{A_{max}} n(A) = 2 + \frac{1}{2} \sum_{A=3}^{A_{max}} \varphi(A). \tag{3}$$

The unity added to the sum takes into account the value $\theta = 0$. In order to better understand the behavior of $\mathcal{N}(A_{max})$ we can use Walfisz approximation for large A_{max}, and assume $A_{max} \approx 2k_{max}$ to get

$$\mathcal{N}(k_{max}) = 1 + \frac{6}{\pi^2} k_{max}^2 + \mathcal{O}\left[k_{max} \left(\log k_{max}\right)^{2/3} \left(\log\log k_{max}\right)^{4/3}\right]. \tag{4}$$

This means that the sexual contact network has $\sim 10^4$ allowed values, and the cattle trade network has $\sim 10^8$ allowed values. Such large number of allowed values in the interval $[0, 1]$ justifies our approximation of treating θ as a continuous variable.

2.2 Temporal stability of the loyalty distribution in cattle and sexual contact networks

Fig. S2 shows the loyalty distributions in all configuration pairs included in the two datasets under study (top, cattle trade network; bottom, sexual contact network). In both networks, distributions are stable in time.

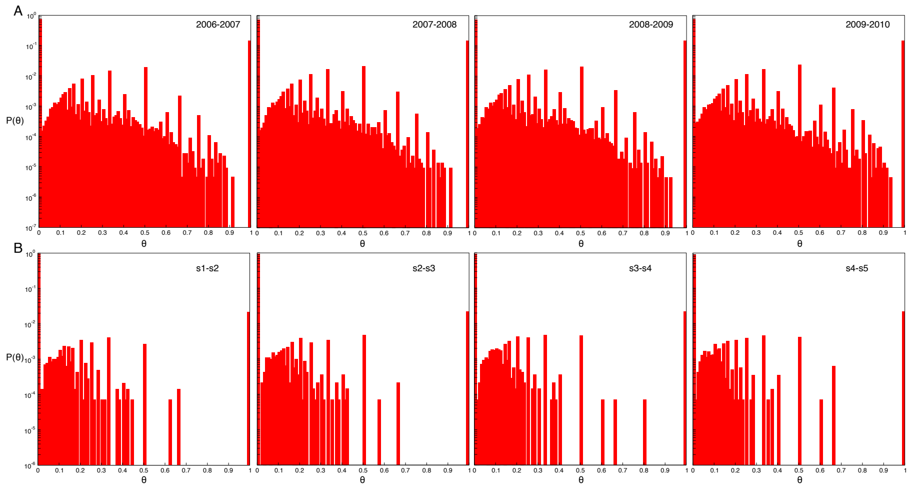

Figure S2: Loyalty distributions for different configurations. (*A*): distributions for cattle network, over the considered time period. (*B*): distributions for sexual contacts network.

2.3 Correlation between loyalty and degree

Degree and loyalty, while not being independent variables, are nonetheless not trivially correlated. Fig. S3 shows the scatter plots between the degree of a node in configuration c and its loyalty for the pair of configurations $c, c+1$, for both networks. For each value of k, θ is found to range over a wide interval. This is clearly visible up to $k \approx 10^2$ for the cattle trade network, and $k \approx 10$ for the sexual contact network. Higher degree nodes are much less frequent, so the statistics becomes poorer and the heterogeneity in θ decreases as k increases. Pearson correlation coefficients are found to be low for both networks (0.04 for the cattle trade network and 0.15 for the sexual contact network), consistently with the observed large variations. They are however significantly larger than the coefficients of the null model: 95% confidence interval of $(-0.002, 0.002)$ and $(-0.006, 0.007)$, for the cattle trade network and the sexual contact network, respectively. This points to a positive, albeit weak, correlation between degree and loyalty. The confidence intervals for the null model are obtained by randomly shuffling several times the sequence of θ's, in order to highlight any spurious correlation with the degree sequence.

3 Loyalty and other similarity measures

We analyze here the relationship between loyalty and other possible measures of similarity of the neighbor structure of a node across time. Firstly we consider a measure introduced as *social strategy* in [1]. In our context, if we call $\tilde{k}_i^{1,c}$ the (in-)degree of node i in the network resulting from the aggregation of snapshots 1 to c, then $i's$ social strategy in those configurations will be computed as $\gamma_i^{1,c} = \tilde{k}_i^{1,c} / \left(\sum_{c'} k_i^{c'}\right)$. $k_i^{c'}$ is as usual the (in-)degree of i in configuration c'. This definition is the same as in [1], except for a normalizing factor c. We make this choice in order to make the comparison with θ more straightforward. The most important qualitative

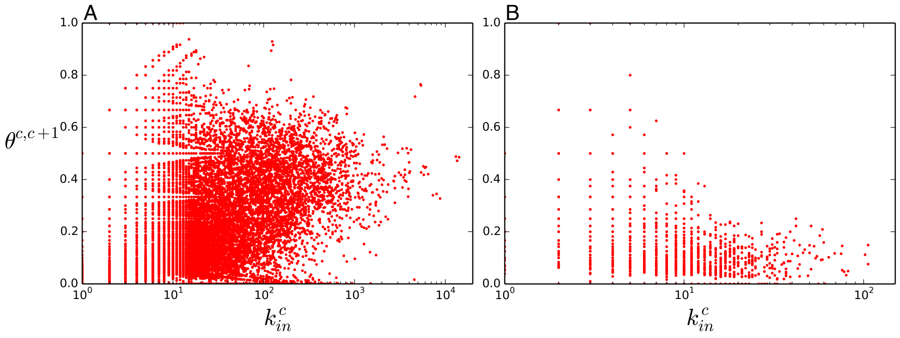

Figure S3: Scatter plots showing degree at configuration c vs loyalty between configurations $c, c+1$. Each point represents a node. (*A*): cattle network. (*B*): sexual contacts.

difference between loyalty and social strategy is that the former is always computed between a pair consecutive snapshots, while the latter typically describes an average behavior computed on several configurations (from 1 to c in our notation). Indeed only in the trivial case of γ computed on just two snapshots, loyalty and strategy are univocally related: $\gamma_i^{1,2} = \frac{1}{1+\theta_{1,2}^i}$. In general, $\gamma_i^{1,c}$ will be a non trivial combination of all the consecutive loyalties $\theta_i^{1,2}, \theta_i^{2,3} \cdots \theta_i^{c-1,c}$ and degrees. Fig. S4A shows the correlation between social strategy in cattle network, computed from 2006 to 2010, and loyalty between 2009, 2010.

We now consider a measure of neighbor similarity derived from Pearson correlation coefficient. This measure is analogous to what is called *adjacency correlation* in [2]. For each node we build two vectors, v_i^c, v_i^{c+1}, of dimension $|\mathcal{V}_i^c \cup \mathcal{V}_i^{c+1}|$, i.e. these vectors will contain an entry for each vector that is neighbor of i in at least one of the two configurations. v_i^c has entries equal to 1 for nodes that are in $\mathcal{V}_i^c$, and zero otherwise, and the same for v_i^{c+1}. We than consider the Pearson correlation coefficient between the two vectors, $\xi^{c,c+1}$. This can be directly related to the loyalty $\theta_i^{c,c+1}$ and the degrees of the node in the two configuration k_i^c and k_i^{c+1} through the formula

$$\xi^{c,c+1} = -\frac{k^c + k^{c+1}}{\sqrt{k^c k^{c+1}}} \frac{1}{1+\theta^{c,c+1}} \sqrt{\left(1+\theta^{c,c+1}\right)^2 \frac{k^c k^{c+1}}{\left(k^c + k^{c+1}\right)^2} - \theta^{c,c+1}} \qquad (5)$$

In the above equation we have omitted the subscript i: $\xi^{c,c+1} = \rho_i^{c,c+1}$, $k^c = k_i^c$ and $\theta^{c,c+1} = \theta_i^{c,c+1}$. Fig. S4B shows the scatter plot $\xi^{c,c+1}$ versus $\theta^{c,c+1}$. We see that, due to the definition of vectors v^c, $\xi \in [-1, 0]$. This formula can be simplified if we need just an average behavior: assuming $k^c = k^{c+1} = k$, where k is the average connectivity, the formula reduces to $\langle \xi^{c,c+1} \rangle = -(1-\theta^{c,c+1})/(1+\theta^{c,c+1})$. From this we get that $\theta = 0$ (no memory) corresponds to $\xi = -1$, while $\theta = 1$ (perfect memory) corresponds to $\xi = 0$.

Finally, we analyze an application of cosine similarity. For each node vectors v_i^c, v_i^{c+1} are built as before. Then cosine similarity between those vectors is defined as $\zeta = v_i^c \cdot v_i^{c+1} / \left(|v_i^c||v_i^{c+1}|\right)$. It can be shown that, like ξ, ζ can be written in terms

of degree and loyalty:

$$\zeta^{c,c+1} = \frac{\theta}{1+\theta}\frac{k^c + k^{c+1}}{\sqrt{k^c k^{c+1}}} \tag{6}$$

The average behavior this time is $\langle \zeta^{c,c+1} \rangle = 2\theta^{c,c+1}/(1+\theta^{c,c+1})$ (see scatter plot in Fig. S4C).

In conclusion, social strategy, being computed on a sequence of more than two configurations, represents a qualitatively different measure with respect to loyalty, albeit the two measures being correlated (see Fig. S4A). On the other hand, both Pearson ξ and cosine similarity ζ can be completely determined in terms of degree and loyalty. Moreover, the mean trend is well modeled by the averaged version of these measure, which discounts degree (see Fig. S4).

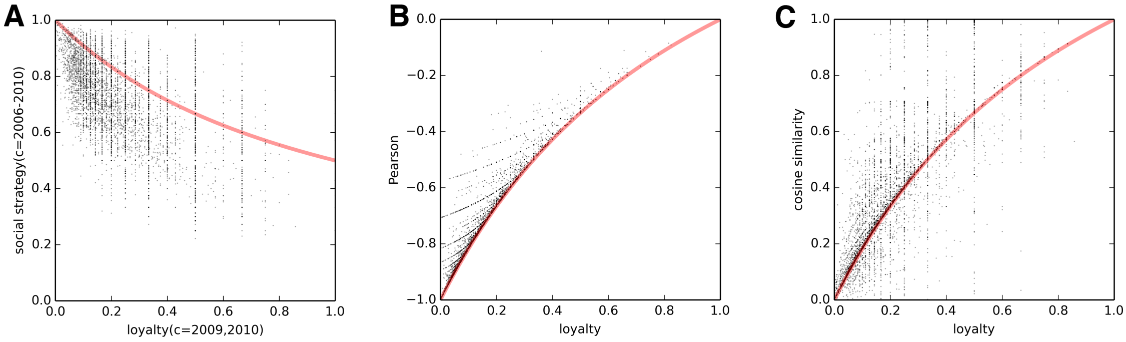

Figure S4: Cattle network: correlation between loyalty and other neighbor similarity measures. *(A)*: scatter plot showing social strategy (γ) computed from 2006 to 2010 vs loyalty between $2009, 2010$. Each point represents a node. The red line represents $\gamma_i^{1,2} = \frac{1}{1+\theta_{1,2}^i}$; Pearson correlation is -0.59. *(B)*: Pearson (ξ) vs loyalty. The red line represents $\langle \xi^{c,c+1} \rangle$. *(C)*: cosine similarity ζ vs loyalty. The red line represents $\langle \zeta^{c,c+1} \rangle$.

4 Modeling infection potentials

Infection potentials π_D and π_L are modeled with a sum of an exponential distribution, to account for the behavior at $\pi \simeq 0$, and a Landau distribution, to mimic the particular asymmetry around the peak. The exact formulation is the following:

$$f(x;\mu,\sigma,r,q) \propto \exp(-qx) + r\int_0^\infty dt\,\sin(2t)\exp\left[-t\frac{x-\mu}{\sigma} - \frac{2}{\pi}t\log t\right]. \tag{7}$$

There are four free parameters: one for the exponential distribution, two for the Landau distribution, and one driving the relative importance of one function with respect to the other. An overall scaling coefficient is fixed by normalization.

5 Robustness of the risk assessment procedure in varying parameters and assumptions

5.1 Threshold ϵ

In the following we examine the behavior of the infection potentials π_D and π_L in varying the value of the threshold. Fig. S5 shows that in the cattle trade network the peak position of π_D increases with ϵ, from 0.3 to 0.6. Such behavior is present in the sexual contact network too, albeit less evident (from 0.3 to 0.5). Unlike π_D, π_L distributions remain stable as ϵ varies. As a result, the probability of a loyal node being infected (π_L) does not depend on the choice of ϵ. The choice of threshold $\epsilon = 0.1$ thus allows to maximize the distance between π_D and π_L distribution while preserving enough statistics for the loyal nodes.

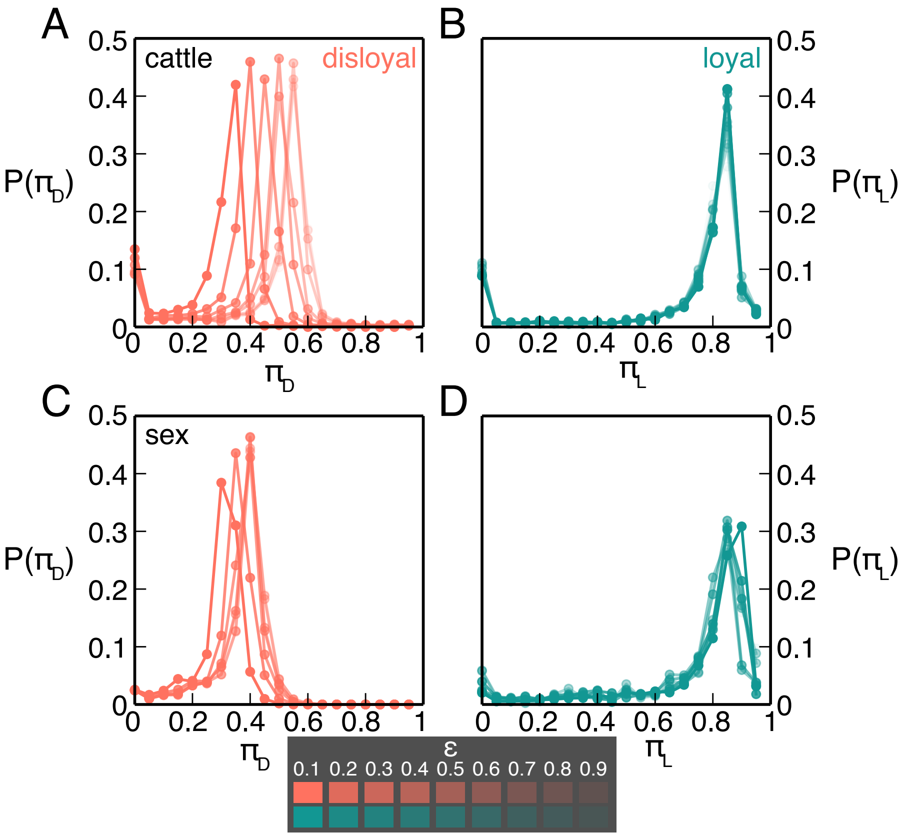

Figure S5: Behavior of infection potentials π_D and π_L as ϵ varies. (*A*),(*C*): π_D curves. (*B*),(*D*): π_L curves. (*A*),(*B*): cattle network. (*C*),(*D*): sexual contacts.

It is important to note that the value of ϵ also affects the transition probabilities T_{DD}, T_{LL} in their functional dependence on the degree (Figure 3C,D of the main text). For each threshold value, such dependence needs therefore to be assessed through a fitting, to be used for the prediction of the loyalty values in the unknown network configuration.

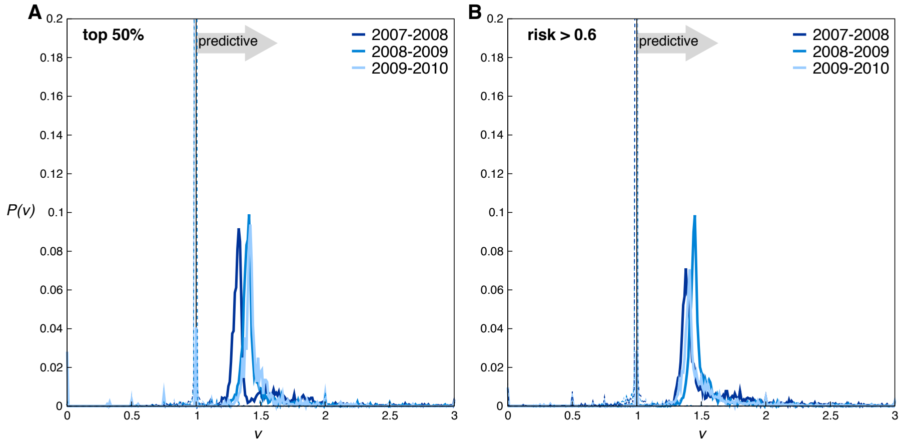

Figure S6: Risk ratio (ν) distribution for cattle network. (A): $\mathcal{I}^c_{s,h}$ as set of top 50% highest ranking nodes. (B): $\mathcal{I}^c_{s,h}$ as set of nodes with $\rho > 0.6$.

5.2 Definitions for the risk ratio ν

In the main paper the risk ratio ν is computed considering the set $\mathcal{I}^c_{s,h}$ of the top 25% highest ranking nodes. Here we explore two different ways of defining this quantity:

- $\mathcal{I}^c_{s,h}$ as the set of the top 50% highest ranking nodes (Fig. S6A);
- $\mathcal{I}^c_{s,h}$ as the set of nodes with epidemic risk $\rho > 0.6$ (Fig. S6B).

Results are reported in Fig. S6 showing the invariance of the observed ν results on this arbitrary choice.

5.3 Definition of the early stage of an epidemic

In the main paper we consider an initial stage of the epidemic up to $\tau = 6$. This choice being arbitrary, it is informed by the simulated time behavior of the incidence curves (see Fig. S7) and the aim to focus on the initial stage of the epidemic.

We also tested a longer initial stage ($\tau = 10$) for the sexual contacts network, to assess the impact of this variation on the obtained results. We obtain distributions of the infection potential, of the relative risk ratio, and of the predictive power showing sharper peaks, however with unchanged peak positions (Fig. S8 for the sexual contact network). Peaks are expected to be sharper, because with $\tau = 10$ a larger fraction of the network is reached by the outbreak. The fact that peak positions do not change, however, reveals that we are able to provide accurate epidemic risks already at the earlier phase of the epidemic ($\tau = 6$), when such information is mostly needed.

5.4 Aggregation time window

The choice of yearly aggregation time in the case of the cattle trade network is informed by its annual seasonal dynamics; the six-months aggregating window for the sexual contact network is instead arbitrary. Here we explore other aggregating

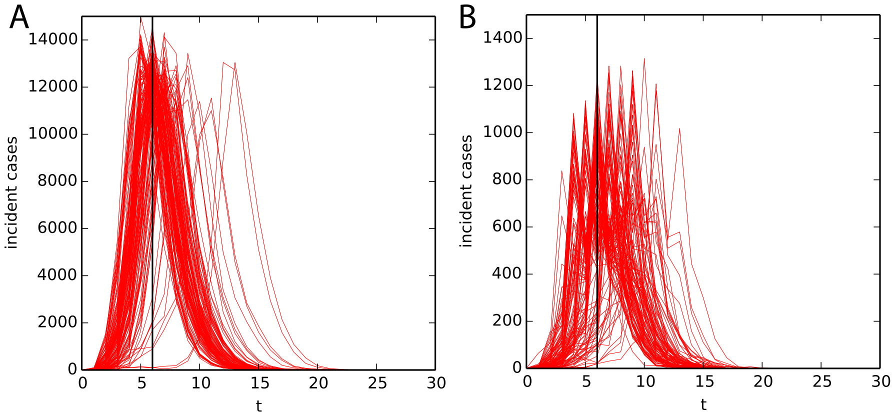

Figure S7: Simulated incidence curves obtained by changing seeding node and network configuration for the cattle trade network (*A*), and the sexual contacts network (*B*). Black line indicates $\tau = 6$.

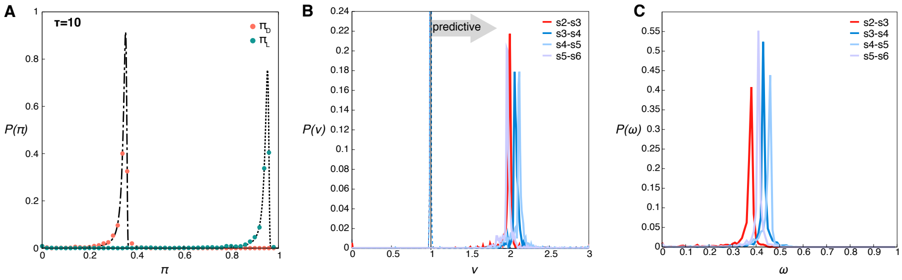

Figure S8: Invasion stage of the outbreak up to $\tau = 10$ for sexual contacts network: distribution of the infection potentials (*A*), the risk ratio ν (*B*)and the predictive power ω (*C*).

windows for both networks to explore the impact they may have on the obtained results.

We consider configurations for the sexual contact network consisting of 3-months aggregation. When calculating the risk ratio and the predictive power (Fig. S9B,D), we find distributions similar to the ones reported in the main text, with unchanged peak positions. The distributions however appear to be noisier, especially as far as ω is concerned, likely induced by the increased sparseness of the network configurations.

We also try a different aggregation time for cattle network: 4-month windows. Risk ratio and predictive power distributions are presented in Fig. S9A,C. We observe that ω is on average quite low: this is likely due to the fact that aggregation windows shorter than one year fail to take into account the seasonal patterns, thus decreasing system memory.

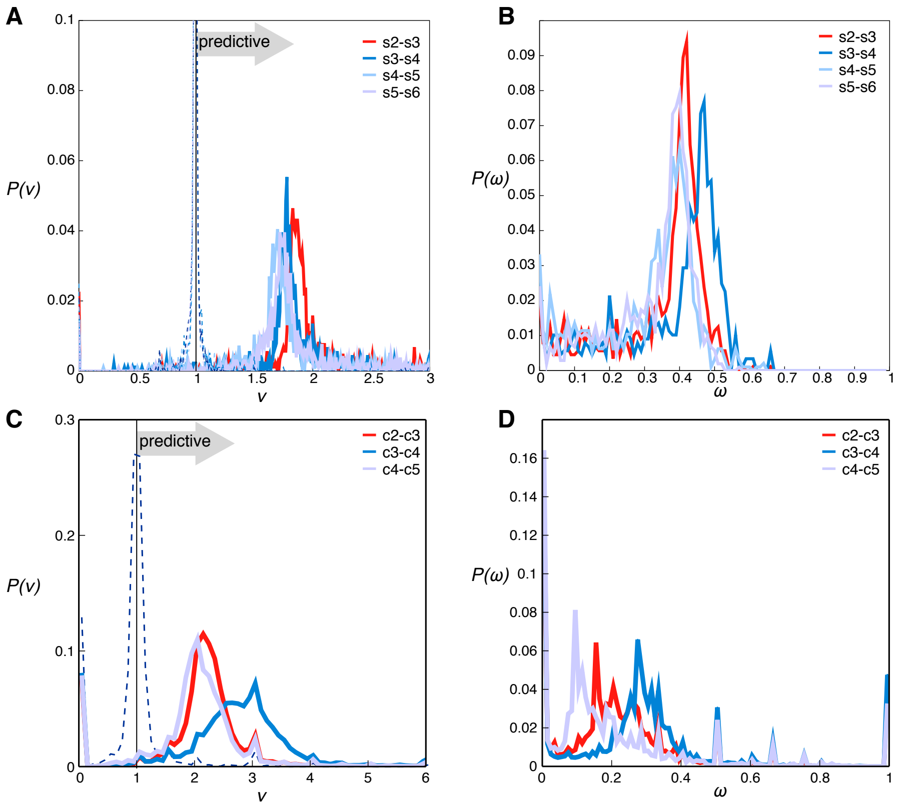

Figure S9: Exploration of different aggregating windows (cattle: 4-month, sex: 3-month). Distributions of risk ratio ν in cattle (A) and sexual contacts (C). Distribution of predictive power ω in cattle (B) and sexual contacts (D).

6 Memory driven model: analytical understandings

6.1 Amount of memory

In the following we analytically quantify the amount of memory in the memory driven model as the probability $f_{c,c+1}$ that a link present in configuration c is also present in configuration $c+1$. This can be expressed as:

$$f_{c,c+1} = (1-d)\left[p_\alpha + \frac{1}{N}\frac{b(1-d)}{b+d}\frac{\zeta(\gamma-1)}{\zeta(\gamma)}\right], \tag{8}$$

where the first term, $(1-d)p_\alpha$, is the probability of remaining active and at the same time keeping a particular neighbor. The second term is the probability of not keeping a neighbor but recovering it with one of the new stubs. ζ is the Riemann ζ-function. $f_{c,c+1}$ can indeed be interpreted as the system memory, as it is a good estimator of the fraction of links that survive from one configuration to the following.

The second term in Eq. 8 is suppressed by $1/N$ and can be disregarded in our case given the large size of the networks ($N = 10^4$). $f_{c,c+1} \approx (1-d)p_\alpha$ therefore provides a first order approximation that correctly matches the numerical results (see Fig. S10A for the comparison).

6.2 Probability associated to zero loyalty

The probability of a node with in-degree h_c having zero loyalty ($\theta_{c,c+1} = 0$) can be computed analytically as

$$P(\theta_{c,c+1} = 0|k_c) = d + (1-d)\,(1-p_\alpha)^{k_c}\,. \qquad (9)$$

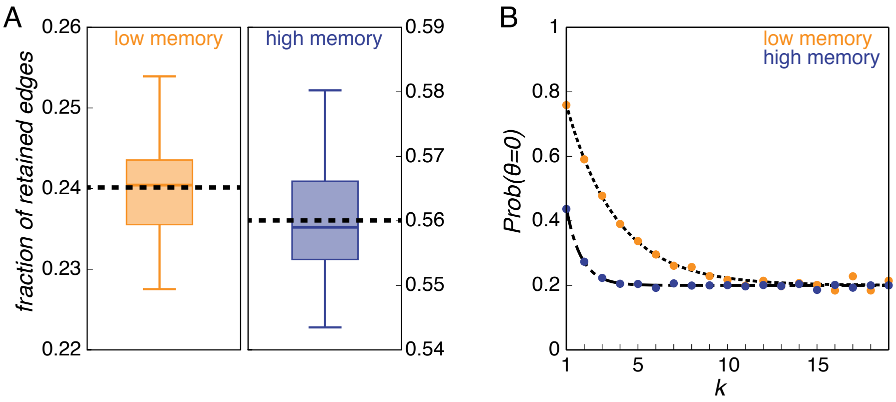

Figure S10: Characterization of the memory driven dynamical model. (*A*): the memory of the system, in terms of the fraction of edges retained from one configuration to the following. Boxplots represent median and quartile positions. The distributions are computed over 50 realizations of the model. Dashed lines represent the theoretical prediction. $p_\alpha = 0.3, 0.7$ for low and high memory, respectively. (*B*): probability for a node with a given in-degree k to be completely disloyal ($\theta = 0$) between two following snapshots. Points represent numerical simulations, while lines show the theoretical estimates.

In Fig. S10B we check this result against numerical simulations.

7 Memory driven model: additional properties

In the main paper the transitions probabilities between loyalty statuses are shown only for the real networks (main paper Fig. 3C and 3D). Here we present them for the memory driven model. Fig. S11 reports these probabilities in case of low and high memory, along with the modeling functions.

In addition, we explore different values of the model parameters and discuss the changes in the network properties. In particular, we explore different values for the probability of becoming active (b) or inactive (d), other than the choice used in main paper ($b = 0.7$, $d = 0.2$). Fig. S12A, S12B, S12C are the equivalent of main paper Fig. 5A, and show the in-degree distribution for different values of b, d in the set $\{0.2, 0.7\}$. $P(k_{in})$ is very robust when changing these parameters, and in all cases follows the slope of the β_{in} distribution. Fig. S12D, S12E, S12F are the equivalent of main paper Fig. 5B, and show the loyalty distributions. We observe that the overall shape is insensitive to parameters change. There is however, a tendency to

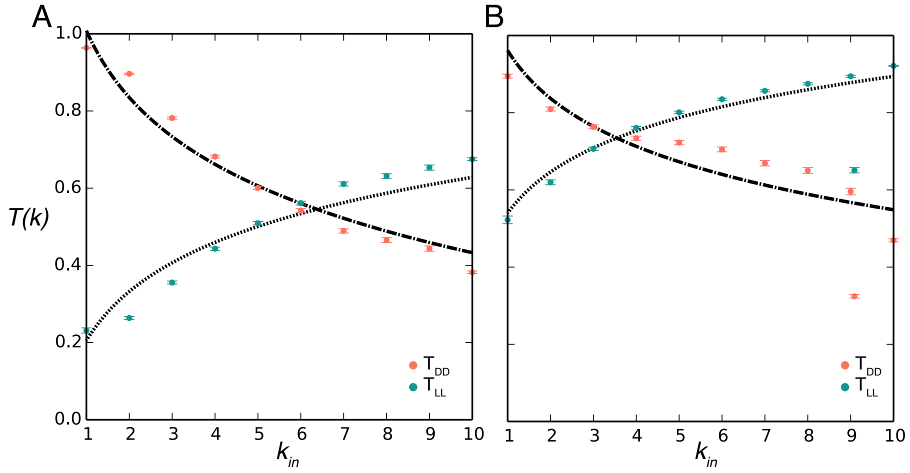

Figure S11: Memory driven model: loyalty transition probabilities between loyal statuses ($T_{LL}(k)$, green) and disloyal statuses ($T_{DD}(k)$, orange) as functions of the degree k_{in} of the node. (*A*): low memory model ($p_\alpha = 0.3$), (*B*): high memory model ($p_\alpha = 0.7$). Dashed lines represent the logarithmic models: $T_{DD}(k) = 1.01 - 0.25 \log k$, and $T_{LL}(k) = 0.20 + 0.18 \log k$ for the low memory; $T_{DD}(k) = 0.96 - 0.17 \log k$, and $T_{LL}(k) = 0.53 + 0.15 \log k$ for high memory. Error bars represent the deviation $\pm \{T(k) [1 - T(k)] / N_k\}^{1/2}$, where N_k is the number of nodes with degree k used to compute $T(k)$. Last value for k: $k = 10$ includes all nodes with degree equal or higher.

have higher θ values for low b, d. This is to be expected, since higher probabilities of going from active to inactive and vice versa mean larger turnover, which leads to lower memory and therefore lower overall loyalty.

8 Validation in the stochastic case

We repeat the analysis reported in the main text by considering a stochastic Susceptible-Infectious approach. Given the same initial conditions, we perform r different stochastic runs, each leading to potentially different outcomes. For each node i, we compute the fraction $f_i(s)$ of runs that node i is infected from epidemics starting from seed s within time step τ. For validation, we need to compare the list $\{\rho_i\}(s)$ of the node epidemic risks computed with our methodology with the list $\{f_i\}(s)$ of the probabilities of actually getting infected. If our estimated risks are reliable, then the two lists need to be correlated, as a higher risk should correspond to a higher probability to get infected. In order to evaluate this, we compute the Pearson correlation coefficients between $\{\rho_i\}(s)$ and $\{f_i\}(s)$, for each possible seed s. The list of these coefficients can then be summarized in a distribution. Fig. S13B and S13D show such distributions for the sexual contact network for two different values of the infection transmissibility (0.75 and 0.85, respectively). In order to check that the correlation coefficients are significantly different from zero, we compute the same distributions after reshuffling the epidemic risks (dashed lines in plots). Fig. S13A

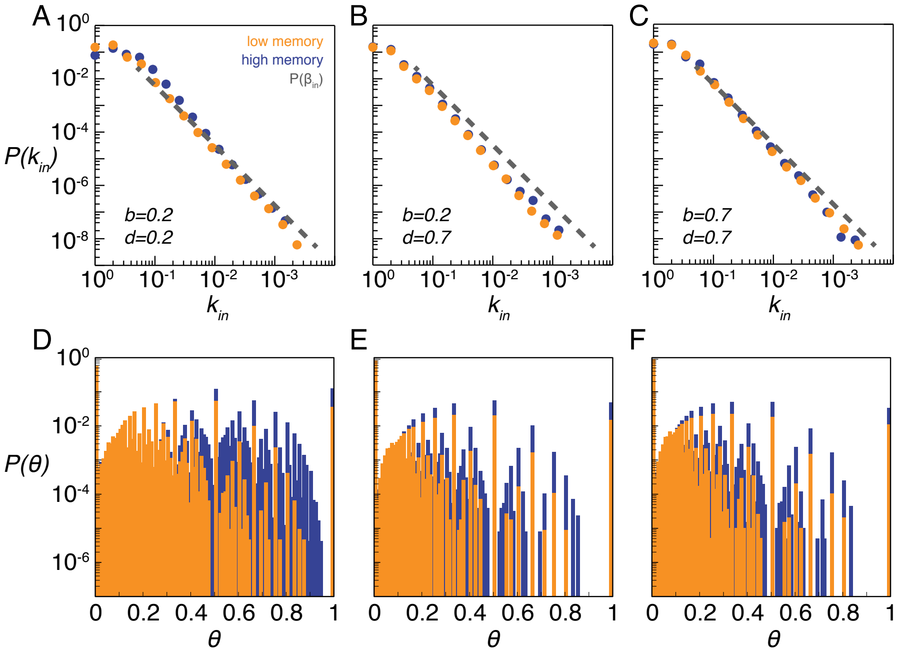

Figure S12: Memory driven model: degree and loyalty distribution when changing the probabilities of becoming active or inactive. (*A*),(*B*),(*C*): in-degree distributions when $(b, d) = (0.2, 0.2), (0.2, 0.7), (0.7, 0.7)$, respectively. (*C*),(*D*),(*E*): loyalty distributions for the same parameter configurations.

and S13C are the equivalent of Fig. 3B in main paper and show that the peak position of the infection potential does not change from the deterministic case. Noise and peak width, however, increase considerably, as well as the probability of having $\pi_D = 0$, and this effect is more pronounced for lower infection transmissibilities.

9 Cattle network: taking into account links weights

Links in cattle network can be assigned a weight attribute in terms of the number of moved animals. These additional data can be included in the modeling of diseases spread, assuming that larger batches have a greater probability of carrying the disease from the source holding, to the destination. This feature is included in the disease model, by assuming a per-animal transmissibility λ. Then, given a movement of w animals, the transmission probability along that link will be $[1 - (1 - \lambda)^w]$ (same approach as in SI of [3]). Loyalty needs to be generalized to the case of weighted network, too. The most straightforward generalization is obtained by considering the quantities in Eq. (2) of main paper $\mathcal{V}_i^{c-1}, \mathcal{V}_i^c$ as multisets (see, for instance, [4]), where each neighbor appears as many times as the weight of the corresponding link. Then the *weighted loyalty* on the weighted network is defined, as before, by Eq. (2) of main paper, using the definitions of multiset union and intersection: $\mathcal{V}_i^{c-1} \cup \mathcal{V}_i^c = \sum_j \max\left(w_{ji}^{c-1}, w_{ji}^c\right)$ and $\mathcal{V}_i^{c-1} \cap \mathcal{V}_i^c = \sum_j \min\left(w_{ji}^{c-1}, w_{ji}^c\right)$, where w_{ji}^c is the weight of the link $j - i$ in configuration c (assuming $w = 0$ if no such link is present). Other choices of similarity between sets of neighbors are

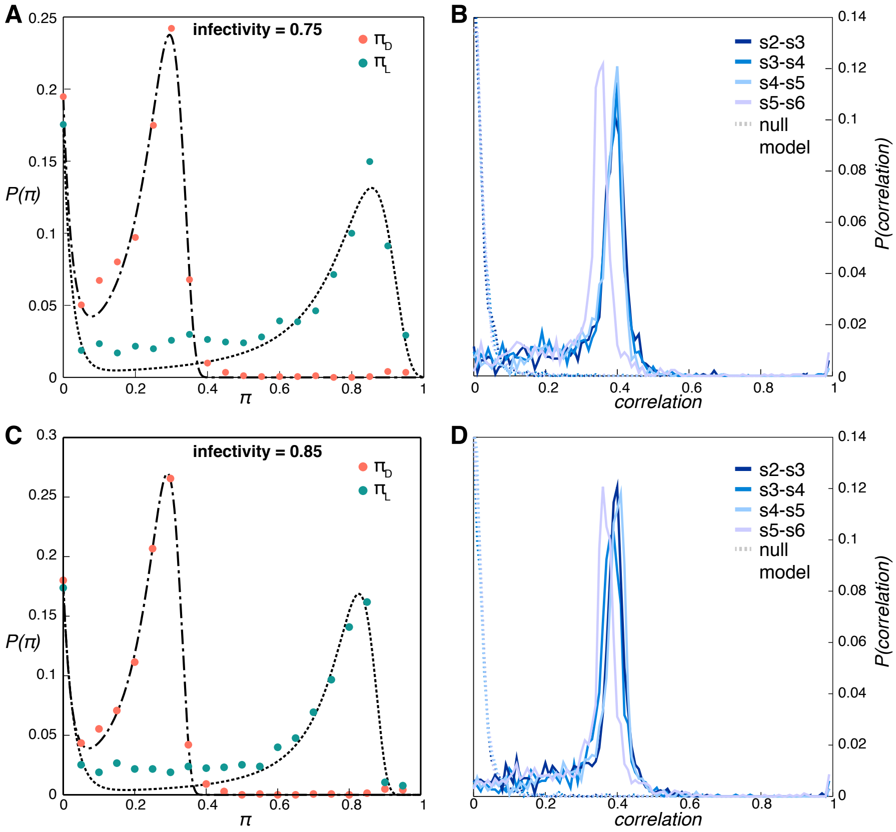

Figure S13: Applying the methodology to sexual contact networks using a stochastic epidemic model. (*A*),(*C*): infection potentials for infectivity 0.75 and 0.85, respectively. (*B*),(*D*): distribution of the Pearson correlation coefficient between the computed epidemic risks and the probability of actually being infected, for infectivity 0.75 and 0.85, respectively. Dashed lines show distributions from the null model.

possible, however this one is the most natural generalization, since it has a very similar distribution to the unweighted loyalty (Fig. S14A), and correlates well with it (Fig. S14B). We now compute the infection potentials and then the epidemic risks, using this new loyalty. We validate the computed risks analogously to what we did in Sec. 8. Results are presented in Fig. S15, showing the generalizability of our approach to the weighted case too.

10 Assessing the robustness of risk based prediction with respect to simple predictors

We have shown that ρ effectively represents the risk of being infected, as shown in the Validation section of main paper. We now show that ρ is a significant improvement in prediction accuracy, with respect to simpler measures, like the degree of a node. From configurations $c-1, c$ of cattle network we compute the risk of being

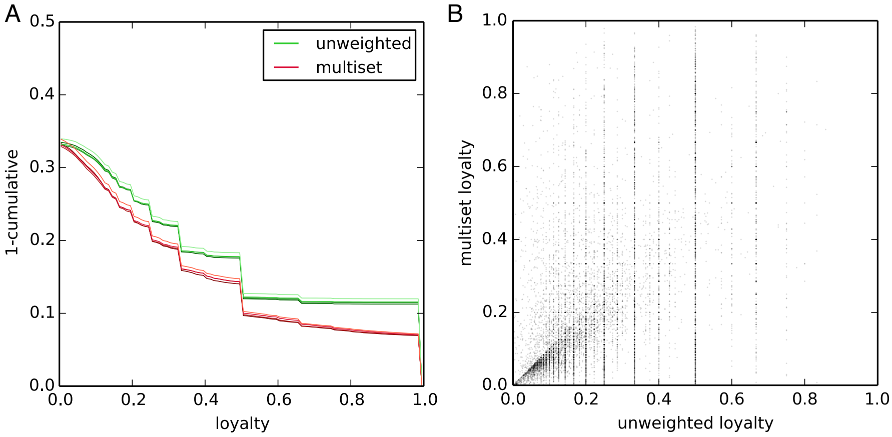

Figure S14: Weighted cattle network: extending the definition of loyalty. *A* shows the cumulative distributions for the unweigthed loyalty (green) and multiset loyalty (red). Different tones of colors refer to different network configurations. *B* Scatter plot correlating the unweighted loyalty and the multiset loyalty. Pearson correlation coefficient is 0.92.

infected at $c+1$: $\rho_i = \rho_i^{c+1}(s)$, as in Eq. (3) of main paper. For each node i for which we can compute ρ_i we then have the binary variable *outcome* indicating if node i is eventually hit by the epidemic in configuration $c+1$. We perform a multivariable logistic regression to check that ρ is actually a predictor for *outcome*, adjusting the in-degree in configuration c: k_i^c. In particular, due to the high heterogeneity of k, we adjust for the log of the degree. Tab. S1 shows the results of the performed regressions. As the crude odds ratios show, both ρ and k^c, on their own, are meaningful predictor of infection in configuration $c+1$. We are however interested in assessing wether our risk is still a predictor, once the effect of knowing the degree is discounted for. The odds ratio for ρ adjusted for degree is still significantly greater than one, meaning that even within nodes of the same degree, nodes at high risk are likelier to get infected. In other words, computing the risk (for which the knowledge of the degree of the node is needed) gives more predicting power than the sole knowledge of degree.

	**crude OR**		**adjusted OR**	
**log(degree)**	2.88	$[2.87, 2.89]$	2.08	$[2.07, 2.10]$
**risk**	4.82	$[4.78, 4.86]$	2.50	$[2.49, 2.51]$

Table S1: Odds ratios of being infected in configuration $c+1$, given degrees in c and computed risks. Crude odds ratios refer to two separate univariate regressions; adjusted odds ratios are obtained through a single multivariate regression. 95% confidence intervals are reported.

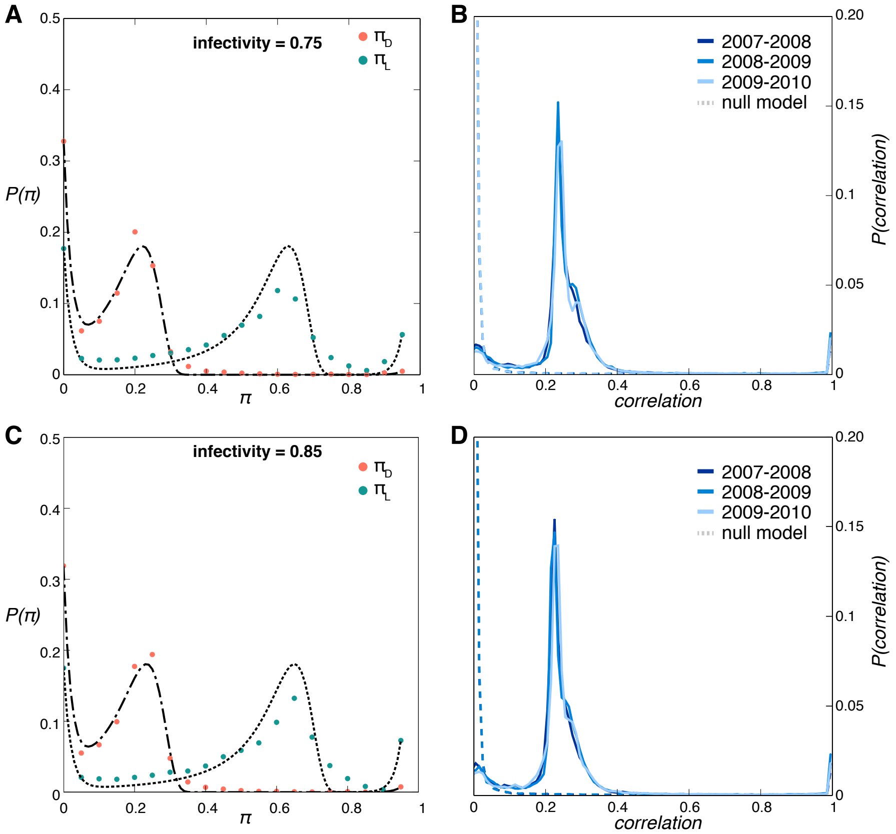

Figure S15: Weighted cattle network: risk prediction computation and validation. (*A*),(*C*): infection potentials for single-animal infectivity 0.75 and 0.85, respectively. (*B*),(*D*): distribution of the Pearson correlation coefficient between the computed epidemic risks and the probability of actually being infected, for infectivity 0.75 and 0.85, respectively. Dashed lines show distributions from the null model.

11 Application to human proximity networks

The main difficulty in applying our methodology to physical proximity networks in human is that generally those networks are much smaller than the ones we have examined, that making it difficult to reach enough statistics to fit the form of infection potentials and transitions probability, and then perform the validation. We show here how we can overcome these impairments and apply successfully our strategy to a network of face-to-face proximity at a scientific conference, collected by the Sociopatterns group [5]. This network records the interactions of 113 nodes during a period of 2.5 days. We split such networks in 30 configurations (corresponding to hourly time steps), and use the first 29 configurations to train our methodology, in order to give predictions on the $30th$. We use this large number of configurations in order to be able to build reliable empirical distributions for the infection potentials and the transition probabilities between loyalty statuses. Once risks are computed as usual, it is not possible, however, to perform the validation as we did for cattle, sexual contacts and memory driven models. This impossibility arises from the

fact that the computed risk ratios are too few to build their distribution. In order to validate our methodology we therefore use the same technique implemented in Sec/ 10: for every node, we compute the odds ratio of being infected in the last configuration, given the knowledge of degree and the computed risk. Results are reported in Tab. S2. Computer risks are strong predictors for infection, even after adjusting for degree. Moreover, unlike cattle network (see Tab. S1), degree alone is not a predictor. Predictive power ω is on average high: median 0.87, with quartiles $Q_1 : 0.69, Q_2 : 0.97$.

	**crude OR**	**adjusted OR**
**log(degree)**	1.16 [1.13, 1.20]	0.95 [0.89, 1.02]
**risk**	11.97 [7.79, 18.4]	22.34 [7.90, 63.3]

Table S2: Odds ratios of being infected in last configuration last, degree and computed risk. Crude odds ratios refer to two separate univariate regressions; adjusted odds ratios are obtained through a single multivariate regression. 95% confidence intervals are reported.

References

[1] Miritello G, Lara R, Cebrian M, Moro E (2013) Limited communication capacity unveils strategies for human interaction. Sci Rep 3, 1950.

[2] Clauset A, Eagle N (2012) Persistence and periodicity in a dynamic proximity network, ArXiv:1211.7343.

[3] Bajardi P, Barrat A, Savini L, Colizza V (2012) Optimizing surveillance for livestock disease spreading through animal movements. J Roy Soc Int (June, 2012).

[4] Stanley R P (1997). Enumerative Combinatorics, Vols. 1 and 2., Cambridge University Press.

[5] Isella L, Stehlé J, Barrat A, Cattuto C, Pinton J-F, Van den Broek Wouter (2011) What's in a crowd? Analysis of face-to-face behavioral networks, Journal of Theoretical Biology 271,1:166-180.
